# Epigenetic and Genetic Integrity, Metabolic Stability, and Field Performance of Cryopreserved Plants

**DOI:** 10.3390/plants10091889

**Published:** 2021-09-13

**Authors:** Min-Rui Wang, Wenlu Bi, Mukund R. Shukla, Li Ren, Zhibo Hamborg, Dag-Ragnar Blystad, Praveen K. Saxena, Qiao-Chun Wang

**Affiliations:** 1 State Key Laboratory of Crop Stress Biology for Arid Areas, College of Life Science, Northwest A&F University, Yangling District, Xianyang 712100, China; wangmr@nwsuaf.edu.cn; 2 State Key Laboratory of Crop Stress Biology for Arid Areas, College of Horticulture, Northwest A&F University, Yangling District, Xianyang 712100, China; 3 Department of Plant Agriculture, Gosling Research Institute for Plant Preservation, University of Guelph, Guelph, ON N1G 2W1, Canada; wenlubi@uoguelph.ca (W.B.); mshukla@uoguelph.ca (M.R.S.); psaxena@uoguelph.ca (P.K.S.); 4 Institute for Agri-Food Standards and Testing Technology, Shanghai Academy of Agricultural Sciences, Shanghai 201403, China; renliaqx@163.com; 5 Division of Biotechnology and Plant Health, Norwegian Institute of Bioeconomy Research (NIBIO), 1431 Ås, Norway; zhibo.hamborg@nibio.no (Z.H.); dag-ragnar.blystad@nibio.no (D.-R.B.)

**Keywords:** cryopreservation, (epi)genetic integrity, field performance, metabolic stability, reactive oxygen species, shoot tips, tissue culture

## Abstract

Cryopreservation is considered an ideal strategy for the long-term preservation of plant genetic resources. Significant progress was achieved over the past several decades, resulting in the successful cryopreservation of the genetic resources of diverse plant species. Cryopreservation procedures often employ in vitro culture techniques and require the precise control of several steps, such as the excision of explants, preculture, osmo- and cryoprotection, dehydration, freeze-thaw cycle, unloading, and post-culture for the recovery of plants. These processes create a stressful environment and cause reactive oxygen species (ROS)-induced oxidative stress, which is detrimental to the growth and regeneration of tissues and plants from cryopreserved tissues. ROS-induced oxidative stresses were documented to induce (epi)genetic and somatic variations. Therefore, the development of true-to-type regenerants of the source germplasm is of primary concern in the application of plant cryopreservation technology. The present article provides a comprehensive assessment of epigenetic and genetic integrity, metabolic stability, and field performance of cryopreserved plants developed in the past decade. Potential areas and the directions of future research in plant cryopreservation are also proposed.

## 1. Overall Developments and Progresses in Plant Cryopreservation

Cryopreservation refers to the storage of biological samples, such as cells, tissues, and organs, in liquid nitrogen (LN) at extremely low temperatures, usually −196 °C. The motive of initial studies was to establish cryopreservation methods for the long-term preservation of plant genetic resources [1,2]. Since the first success of plant cryopreservation using the two-step freezing of mulberry (*Morus alba*) by Sakai [1], considerable progress has been made in the field over the past 65 years [3,4,5,6]. Successful cryopreservation studies resulted in a number of technical advancements, including the development of various cryopreservation methods, such as vitrification [7,8,9], encapsulation-vitrification [9,10], droplet-vitrification [11], encapsulation-dehydration [10,12] and the use of cryo-plates [13,14]. Of these, the droplet-vitrification method, described by Panis et al. [11], is noteworthy as it has largely helped to remove the genotype-specific barriers, which had impeded the potential application of cryopreservation technology across diverse species. These technical refinements led to the successful cryopreservation of a wide range of genotypes in many plant species, such as *Malus* [15], *Vitis* [16,17], and *Lilium* [18]. The cryopreservation technology is considered an ideal strategy for the long-term preservation of plant genetic resources [3,4,5,6].

Diverse plant species have been successfully cryopreserved, including tuber crops [19,20], fruit crops [15,21], ornamental species [22], medicinal herbs [23], and forest species [24], as well as endangered and endemic plants [25,26,27,28,29]. Cryo-banks using shoot tips have been set up in several countries for economically important plant species [3,4,20,23,30,31]. Studies have also advanced in the evaluation of the performance of cryo-derived plants when they are re-introduced from laboratories to their natural habitat [25,28,32].

Cryopreservation has been shown to retain or even promote the regenerative capacity of embryogenic tissues, which were widely used in genetic transformation in various plant species [31,33]. The use of cryopreserved embryogenic tissues for genetic transformation improved the transformation efficiency and regeneration frequency of transformed plant tissues [34]. Cryopreservation was shown to maintain the transgenes in transformed materials, providing a safe and reliable strategy for the long-term preservation of transgenes [31,33,34]. These studies demonstrated the usefulness of cryobiotechnology in plant genetic engineering.

Shoot tip cryotherapy, which was refined based on shoot tip cryopreservation, has been established as a novel method for the efficient eradication of plant pathogens, including viruses, phytoplasma, and bacteria [31,35,36]. Cryopreservation has also been successfully applied to cryopreservation of plant viruses and viroids in cryopreserved shoot tips [37,38,39], opening a new avenue for the long-term preservation of plant obligate pathogens, which are otherwise difficult-to-preserve for long periods with the traditional methods. Thus, cryobiotechnology offers the dual advantage in the preservation as well as eradication of plant pathogens [38,40].

A number of plant species have been threatened and are currently facing extinction, mainly by anthropogenic processes, such as deforestation, land clearing for agricultural, industrialization, and urbanization, as well as through plant diseases and pests [41,42]. Global warming has further worsened the situation. A recent report warned that about 21% of total global plant species (approximately 390,900 plants) are at risk of extinction [43]. Therefore, special attention should be paid to the cryopreservation of these endangered and rare species [41,42,44,45]. However, studies on cryopreservation of endangered and rare plant species are far behind those of horticultural species, ornamental plants, tuber crops, and forest species [6,15,19,20,21,22,23,24,25,26,27,28,29,41,42].

## 2. Major Concerns in Recovery of Plants from Cryopreserved Tissues

**Figure 1:**
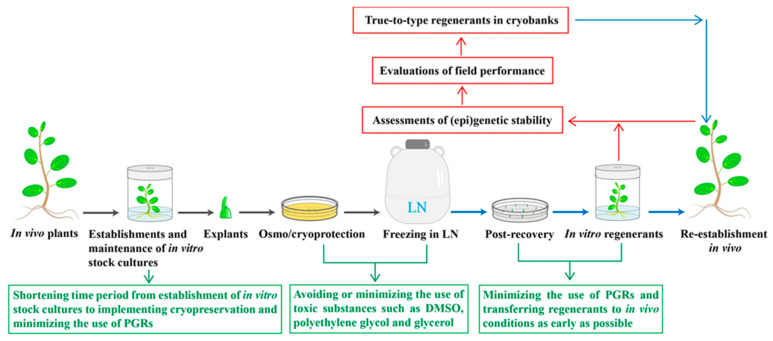
General cryopreservation procedures (black arrows), post-culture for recovery and re-establishment of plants in vivo (blue arrows), assessments of (epi)genetic stability and evaluations of field performance in cryo-derived regenerants/plants (red arrows), and measures taken to ensure (epi)genetic stability and true-to-type regenerants/plants recovered after cryopreservation (green arrows). DMSO, dimethyl sulfoxide; LN, liquid nitrogen; PGRs, plant growth regulators.

For the preservation of plant genetic resources, it is necessary to not only ensure viability and quality of the preserved plants, but to also ensure the true-to-type status of the regenerants (Figure 1). Shoot tips or buds are able to develop into plants that are generally genetically stable and identical to the source plants, and are therefore preferred over other tissues, such as cell suspensions, embryogenic tissues, and callus for preserving plant genetic resources [6,31,46,47,48].

In vitro tissue culture has become an integral part of the cryopreservation technology currently used for the establishment and maintenance of stock cultures, and the post-culture process for the recovery of shoot tips, cell suspensions, and embryogenic tissues, in a number of plant species [6,31,46,48]. However, in vitro tissue cultures are prone to somaclonal and (epi)genetic variations [49,50,51,52]. It is well documented that in vitro tissue culture imposes stressful conditions and induces the generations of reactive oxygen species (ROS), thus resulting in ROS-induced oxidative stress [49,50,51]. Cryopreservation requires the use of plant tissue culture methods in several essential steps, including the excision of explants, preculture, osmo- and cryoprotection, dehydration, freeze-thaw cycle, unloading, and the re-initiation of cultures for plant recovery. All of these steps have been shown to cause ROS-induced oxidative stress [4,26,47,53,54,55].

ROS are highly reactive and toxic by-products of aerobic metabolism. ROS include the superoxide anion, hydrogen peroxide, and hydroxyl radicals, all of which have inherent reactivity to different biological targets, playing a dual role of inducing cellular toxicity, while also serving as signaling transduction stimuli in plant responses [56,57]. The ROS-induced oxidative stresses have proven to induce the somaclonal and (epi)genetic variations in in vitro-derived regenerants [49,50,51,58] and in cryo-derived regenerants [4,26,47,53,54,55]. Therefore, it is necessary to assess and monitor the (epi)genetic integrity and field performance of cryo-derived regenerants. Literature (up to 2010) on relevant aspects of such genetic variations in plants originating from in vitro and cryopreserved cultures was comprehensively reviewed previously [31,41,42,47,53,54,55].

The present review focuses on the advances made in the past decade in the assessment and improvement of the (epi)genetic integrity, metabolic efficiency, and field performance of cryo-derived plants. Research areas and applications that may benefit from further studies on plant cryopreservation are also discussed. These efforts would certainly promote further studies on cryopreservation of endangered and rare plant species.

## 3. Assessments of Epigenetic and Genetic Integrity

### 3.1. Epigenetic Integrity

Epigenetics refers to heritable changes in gene expression that is not associated with changes in the underlying DNA sequence [59]. Major types of epigenetic changes include DNA methylation, histone modification, and changes in chromatin structure [60]. DNA methylation has been widely used for the assessments of epigenetic integrity in cryo-derived plants [47,53,54,55] and methylation-sensitive amplified polymorphism (MSAP) is the most frequently used marker for the assessments of epigenetic stability in plant populations of cryogenic origin.

Hao et al. [61,62,63] were the first to assess epigenetic stability in cryo-derived regenerants. Changes in DNA methylation were detected in plants recovered from the cryopreserved shoot tips of *Malus pumila* “M2” and *Fragaria gracilis* “Joho” [61,63], and somatic embryogenic calli of *Citrus sinensis* “Newhall” [62]. Some representative examples of the use of DNA methylation-induced changes in cryopreservation research in the past decade are listed in Table 1.

Peredo et al. [64] assessed epigenetic integrity in *Humulus lupulus* plants recovered from shoot tips cryopreserved for three years. Changes in DNA methylation were detected in 36% of the loci. Further analysis showed that only 2.6–9.8% of the changes were induced by cryopreservation procedure and the rest were attributed to the in vitro culture processes. In vitro culture-induced DNA methylation was also found in the regenerants recovered from cryopreserved *Carica papaya* shoot tips [65] and *Theobroma cacao* somatic embryos [66]. DNA methylation levels were higher in in vitro stock somatic embryos of *Theobroma cacao* than those regenerated after cryopreservation [66]. Therefore, the manipulations of in vitro culture procedures, particularly for regeneration via somatic embryogenic, are needed to reduce the probability of alternations in DNA methylation.

Alternations in DNA methylation varied with cryogenic steps and cryopreservation durations: 0.12% following PVS2 treatment, 0.12% after 2 h of cryo-storage, and 5.5% after 10 years of cryo-storage in *Wasabia japonica* [67]. Variations in DNA methylation varied among three *Ribes* species, including *R. ciliatum*, *R. sanguineum* and *R. nigrum* [68], and *Humulus lupulus* [64], and *Carica papaya* genotypes [65] subjected to cryopreservation. The assessment of epigenetic stability in the regenerants of the same *Carica papaya* genotype Z6 following cryopreservation detected higher levels of DNA methylation in DNA extracts pooled from mixed leaves of several plants [65] than in those from single leaves of single plants [69]. These data indicated that variations in DNA methylation differed among clones resulted from the same cryopreservation experiment.

Johnston et al. [68] reported DNA methylation in regenerants recovered from *Ribes ciliatum* shoot tips cryopreserved by encapsulation-dehydration. They found that DNA methylation initially occurred during sucrose preculture and progressively increased during successive steps of the encapsulation-dehydration protocol. However, DNA methylation values were similar between the controls and the regenerants recovered from cryopreserved shoots after 18–20 weeks of post-recovery, including two subculture cycles. These data indicate that cryopreservation-mediated DNA methylation is a reversible epigenetic mechanism [68]. Such reversible epigenetic mechanism has also been noted in the regenerants recovered from cryopreserved shoot tips of *Carica papaya* [65], *Mentha × piperita* [70], and *Actinidia chinensis* var. *deliciosa* [71], and from the cryopreserved somatic embryos of *Bactris gasipaes* [72] and *Theobroma cacao* [66]. As speculated by Harding et al. [54], changes in DNA methylation may be an adaptive response to oxidative stress induced during cryopreservation. Therefore, once the stress is removed, DNA demethylation occurs and the regenerants can gradually revert to the normal DNA status.

In contrast with the studies addressed above, no marked alternations in DNA methylation were found in the regenerants recovered from *Quercus robur* plumules cryopreserved by desiccation [73], *Solanum tuberosum* plants from shoot tips preserved with dimethyl sulfoxide (DMSO)-droplet method and cryo-stored for nearly 7 years [74], *Wasabia japonica* plants from vitrified shoot tips cryo-stored for 10 years [68], and *Gentiana cruciata* regenerants from embryogenic cell suspensions cryopreserved by encapsulation-dehydration [75].

**Table 1:** Some examples from the past decade of epigenetic integrity assessments by DNA methylation in regenerants recovered after cryopreservation.

Plant Species	Explants	Cryopreservation Method *	Molecular Methods **	DNA Methylation (%)	Causes	Reference
*Actinidia chinensis* var. *deliciosa*	Shoot tips	Drop-vitri	MSAP	1.6 and 12.8	Cryoprocedures and in vitro cultures	[71]
*Bactris gasipaes*	Somatic embryos	Drop-vitri	The global DNA methylation	25.2–29.7	Cryoprocedures	[72]
*Carica papaya*	Shoot tips	Vitri	AMP	0–0.22	Genotypes and cryoprocedures	[65]
*Gentiana*	Shoot tips	Encap-dehy	MSAP	16.61–16.88	in vitro culture	[75]
*Mentha* × *piperita*	Shoot tips	Encap-dehy	MSAP	17.1–32	Cryoprocedures	[70]
*Quercus robur*	Seed plumules	Desiccation	The global DNA methylation	8.7–11	Cryoprocedures	[73]
*Solanum tuberosum*	Shoot tips	DMSO droplet	MSAP	0.9	Cryoprocedures and in vitro cultures	[74]
*Theobroma cacao*	Somatic embryos	Encap-dehy	MSAP	3.6	Cryoprocedures	[66]
*Wasabia japonica*	Shoot tips	Vitri	MSAP	0.12–5.5	Cryoprocedures	[67]

* Dehy, dehydration; DMSO, dimethyl sulfoxide; Drop, droplet; Encap, encapsulation; Vitri, vitrification. ** AMP, amplified DNA methylation polymorphism; MSAP, methylation sensitive amplified polymorphism.

### 3.2. Genetic Integrity

Molecular markers were widely used for assessments of genetic stability in cryo-derived plants, including random amplified polymorphic DNA (RAPD), inter-simple sequence repeats (ISSR), amplified fragment length polymorphism (AFLP), and single sequence repeats (SSR) [53,54,55]. The RAPD amplifies some areas of the genome and screens a low fraction of the genome, whereas the ISSR amplifies DNA segments between two microsatellite regions [54]. The AFLP technique is based on the selective amplification of restriction fragments from a total digest of genomic DNA [76], and the SSR can detect differences in the length of a particular locus [77,78]. Since different DNA markers detect polymorphisms in different genomic regions, use of more than one molecular marker would provide more reliable results of genetic integrity assessments [31,79]. Therefore, most of the studies conducted in the past decade employed more than one molecular marker for assessments of genetic stability in the regenerants recovered from cryopreservation. Flow cytometry (FCM) was usually used for assessments of DNA ploidy levels in cryopreserved plants [80,81,82]. 

Krajnáková et al. [83] reported some changes in the RAPD profiles of *Abies cephalonica* embryogenic cells after six years of cryopreservation. However, proliferation and maturation abilities were maintained in the cryopreserved cells. Applying microsatellite and sequence-related amplified polymorphism (SRAP) for assessments of the genetic stability in the cryo-derived plants of *Hedeoma todsenii* after 13 years of cryo-storage, Pence et al. [84] did not find any DNA variations in the same genotypes, but found an average of 10.4% variation between the replicate samples. Genetic variations were observed in the regenerants recovered from cryopreserved shoot tips of *Chrysanthemum morifolium*, with 40% and 6% of polymorphic bands detected by AFLP and RAPD, respectively [85]. Further analysis found that the genetic variations detected by RAPD were induced in the sucrose preculture step (0.3 M sucrose at 5 °C for 3 days), while those detected by AFLP were in the cold-hardening of the in vitro stock shoots (10 °C for 3 weeks). Freezing in LN induced the highest levels of genetic variations analyzed by both RAPD and AFLP [85]. Applying RAPD to assessments of genetic stability in the regenerants recovered from cryopreserved shoot tips of *Mentha × piperita*, Martín et al. [86] reported that genetic stability varied with genotypes and cryoprocedures: 97% by droplet-vitrification and 87% by encapsulation-dehydration in “MEN 198” (stable genotype), and 80% by droplet-vitrification and 24% by encapsulation-dehydration in “MEN 186” (sensitive genotype). In the analysis of the genetic stability by FCM, RAPD, and ISSR in the cryo-derived plants of three *Chrysanthemum chimeric* cultivars grown in greenhouse conditions, Kulus et al. [80] reported that FCM did not detect any differences in DNA ploidy levels among the three cultivars. RAPD detected no polymorphic bands in “Richmond” (a solid mutant), but detected 7.8% and 3.2% polymorphic bands in “Lady Orange” and “Lady Salmon” (periclinal chimeras). ISSR markers detected 15% polymorphic bands in “Lady Orange” and no polymorphic bands in “Lady Salmon” and “Richmond” [80].

González-Benito et al. [87] tested the effects of adding vitamin E in the pretreatment medium on cryopreservation and genetic stability in “MEN 186” and “MEN 198”. They found that although it did not have significant effects on recovery, vitamin E improved the genetic stability in the regenerants recovered after cryopreservation, particularly in the sensitive genotype “MEN 186”.

In the cryopreservation of *Rubus grabowskii* shoot tips, Castillo et al. [88] reported that the SSR did not detect any polymorphic bands in the cryo-derived regenerants immediately after cryopreservation and those that were subcultured in vitro for seven months after the recovery. The AFLP did not detect any polymorphic bands in the cryo-derived regenerants immediately after cryopreservation, but detected polymorphic bands in the cryo-derived shoots that were subcultured in vitro for seven months. However, when these in vitro cultured shoots were re-established in the field conditions, polymorphic bands were no longer detected [88]. These results indicated that there might be a transitory phase of the polymorphism when the cryopreserved plants were transferred to the field conditions. Similar results were also found in the cryo-derived plants of *Abies cephalonica* [89] and Carica papaya [65]. Nevertheless, further studies are needed to verify these findings of varied polymorphism in plants from cryopreserved tissues.

A number of studies showed that cryopreservation did not cause or caused minor changes in genetic stability of the regenerants. Genetic stability assessments in the regenerants of *Wasabia japonica* following shoot tip cryopreservation detected only 0.27%, 0.95%, and 2.2% variations in AFLP profiles in the samples following exposure to PVS2, cryo-storage in LN for 2 h, and for 10 years, respectively [67]. RAPD and ISSR did not detect any polymorphic bands in the plants recovered from cryopreservation of *Passiflora pohlii* nodal segments [90]. The maintenance of genetic stability was reported in the regenerants recovered after shoot tip cryopreservation in various plant species, including tuber crops such as *Solanum tuberosum,* analyzed by ISSR and FCM [91] and by AFLP and ISSR [79]. The ornamental plant analyses included *Oncidium flexuosum* by FCM [92], *Chrysanthemum morifolium* by SSR and FCM [82] and RAPD and ISSR [93], *Argyranthemum* by AFLP and ISSR [94], *Torenia fournieri* by FCM and ISSR [95], and *Pleione bulbocodioides* (protocorm-like bodies) by ISSR [96]. Similar results were obtained in vegetable crops, such as *Phaseolus vulgariss* (seeds) by SSR [97], *Asparagus officinalis* (rhizome buds) by EST-SSR and FCM [98], and *Allium* by SSR [81], AFLP and ISSR [99]. Examples of analyses of other crops included fruit trees, such as *Musa* (suck meristem) by SSR [100], *Malus* by FCM and ISSR [101,102], *Vaccinium corymbosum* by RAPD and ISSR [103,104], *Vitis* by RAPD and ISSR [17], and *Actinidia chinensis* by AFLP and ISSR [71], and medicinal species, such as *Rabdosia rubescens* by FCM and SRAP [105] and *Bacopa monnieri* by RAPD [106].

Thus far, there were only a few studies that assessed the genetic stability in pathogen-free plants recovered after shoot tip cryotherapy. FCM did not detect any variations in ploidy levels in Chinese jujube plants (*Ziziphus jujuba*), free of Jujube witches’ broom phytoplasma [107], and artichoke plants (*Cynara scolymus*), free of artichoke latent virus, produced by shoot tip cryotherapy [108]. The SSR and AFLP did not detect any polymorphic bands in cryo-derived potato plants (*Solanum tuberosum*) free of potato leafroll virus, potato virus S, and potato virus Y [109].

Cryopreservation was reported to maintain the *NPTII* and *GUS* genes in transgenic *Oryza sativa* protoplasts [110], *hCTlA4Ig* in the transgenic cell suspensions of *O. sativa* [111], *Escherichia coli heat labile enterotoxin* (LT) protein in the transgenic cells of *Nicotiana tabacum* [112], and *npt II* and *Gus* genes in the transgenic sweetgum (*Liquidambar*) embryogenic cultures [113]. Similar examples of genes maintained in other species included the *GUS* gene in the transgenic *Citrus* callus [114], *npt II* gene in the transgenic shoots of *Betula pendula* [115] and *Populus tremula × P*. *tremuloides* [116], human serum albumin in the transgenic BY-2 cell cultures [117], *uidA* gene in the transgenic plants of *Castanea sativa* [118], and the Cry 1Ab in the transgenic plantlets of *Torenia fournieri* [95]. The successful cryopreservation of transgenes provides a safe and reliable strategy for long-term preservation of the transgenic plant materials, which otherwise may be lost by preservation through in vitro cultures and environmental contamination or gene flow by preservation in vivo [33,34]. Some examples of genetic integrity assessments in cryo-derived regenerants are listed in Table 2.

## 4. Metabolic Stability

For plant species that contain special biochemical compounds, the assessment of metabolic abilities is an important issue in cryo-derived plants. *Chrysanthemum morifolium* “Hangju” contains valuable biochemical compounds, such as anthocyanins and carotenoids, and has long been used as a medicine in China [93]. Applying high performance liquid chromatography (HPLC) for quantitative analyses of biochemical compounds in the cryo-derived plants grown in greenhouse conditions, Bi et al. observed no differences in the levels of the five selected biochemical compounds produced between the cryo-derived plants and in vitro-derived plants in the control [93]. No differences were found in the contents of anthocyanins and carotenoids in the inflorescences of the cryo-derived plants and the control of three *Chrysantemum chimeric* cultivars grown in greenhouse conditions, except for reduced chlorophyll contents found in the cryo-derived plants [80]. In *Bacopa monnieri*, HPLC analysis detected no differences in the level of bacoside A, a functional biocompound in *Bacopa monnieri*, in the cryo- and in vitro-derived (control) plants [106]. More recently, Wang et al. [99] observed no significant differences in the levels of carbohydrates and flavanols in bulbs produced by cryo- and in vitro-derived shallots plants grown in greenhouse conditions. These results indicate that metabolic stability can be maintained in the plants derived from cryopreserved tissues.

## 5. Field Performance

### 5.1. Seed Germination and Seedling Growth

Seed conservation provides a useful and relatively easy strategy for preserving genetic resources in seeded plants [41,130]. Three categories of seed storage behavior are generally recognized among species: orthodox, intermediate, and recalcitrant [41,130]. Seeds of most species belong to the orthodox category, and can be dried to low water contents and thus stored at low temperature for extensive periods [4,26,29,130]. Intermediate seeds can withstand partial dehydration, while recalcitrant seeds are sensitive to dehydration. Therefore, they cannot be stored under the desiccation and low temperature conditions for a long period of time [4,26,29,41,130]. The intermediate and recalcitrant seeds contain numerous important tropical and tropical rain forest species [26,29,130]. Cryopreservation is the only technique available for long-term germplasm preservation of these two categories of seeds [4,26,29,130]. There were several studies conducted over the past decade on seed germination and seedling growth in cryopreserved seeds.

No significant differences in seed germinations were obtained between cryopreserved and non-cryopreserved (control) seeds in *Phaseolus vulgaris* [97,131], *Solanum lycopersicum* [132], and *Zea mays* [133]. However, reduced seed germinations were reported in the cryopreserved seeds of *Zea mays* and *Glycine max* [133,134]. In the study of cryopreservation of *Solanum lycopersicum* seeds, Zevallos et al. [135] reported that cryopreservation increased the germination percentage of cryopreserved seeds at day 5 of germination, albeit with no significant differences at day 7. Increased seed germinations were also observed in the cryopreserved seeds of *Teramnus labialis* at 7 and 28 days of germination [136]. The increased seed germinations were attributed to the breaking of physical dormancy by increased malondialdehyde levels induced during cryopreservation [97,132,135,136].

More than 90% and 94% seedlings from the cryopreserved seeds of *Oncidium flexuosum* [92] and *Hibiscus sabdariffa* [137], respectively, survived after transfer to greenhouse conditions. The morphology of the seedlings developed from cryopreserved seeds were similar to those from the control in *Phaseolus vulgaris* [97,131], *Solanum lycopersicum* [135], *Zea mays* [134], *Glycine max* [134], and *Hibiscus sabdariffa* [137]. Seedling growth, measured by the fresh weight of roots, stem, and leaves, was markedly delayed in cryopreserved seeds of *Zea mays* [133]. Seedling growth, including plant height and fresh and dry mass, was greater in seedlings recovered from cryopreserved seeds than in those from the control during the four-week growth [136]. No differences were found in vegetative growth, including shoot length, number of leaves, number and length of roots, and fresh and dry weight, between the cryo-derived and the control seedlings in *Oncidium flexuosum* [92]. Evaluating vegetative growth and grain production in cryo-seed-derived plants of *Phaseolus vulgaris*, Cejas et al. [123] did not find significant differences in all parameters tested, including the number of stem internodes, plant height, fruit number, grain number per plant, and weight per grain between the cryopreserved and the control seeds. In addition, Cejas et al. [131] reported that seed cryopreservation decreased Cu, Cd, and Na uptake, and increased the absorption of B and Al in the cryo-derived seedling (10 days old) of *Phaseolus vulgaris*.

### 5.2. Field Performance of Cryopreserved Plants

Field performance is critical for evaluating the true-to-type cryopreserved plants in comparison to the source plants (Figure 1). Similar survival percentages and vegetative growth were obtained for the cryo- and in vitro-derived (control) plants of *Actinidia chinensis* var. *deliciosa* after their re-establishment in greenhouse conditions [71]. Agrawal et al. [100] compared the field performance of cryo-derived, micropropagated, and field sucker-propagated *Musa* plants and found that greater than 90% of the cryo-derived and in vitro micropropagated plants survived and were established in field conditions. Vegetative growth (plant height and leaf number) and reproductive growth (flowering and fruit production) were similar among the three sources of plants [100]. Vegetative growth patterns, and morphologies of leaves and flower production, were identical in the cryo- and in vitro-derived plants of *Torenia fournieri* [95] and *Chrysanthemum morifolium* [88] when grown under greenhouse conditions. Wang et al. [99] reported that there were no significant differences in rooting, vegetative growth, and bulb production between the cryo- and in vitro-derived plants of *Allium cepa* var. *aggregatum* when grown in the greenhouse.

Zhang et al. [94] reported that, although root formation and vegetative growth in cryo-derived plants was reduced to a certain degree, the quantity and quality of the flowers were similar in both the cryo- and in vitro-derived plants of perennial ornamental species *Argyranthemum* grown in greenhouse conditions. Vegetative regrowth at the early stage was lower in cryo-derived plants of *Chrysanthemum morifolium* grown in the greenhouse [93] and *Solanum tuberosum* cultured in vitro [91] than their corresponding controls. Vegetative growth markedly increased, however, in the cryo-derived plants of *Solanum tuberosum* after 6 months of in vitro culture [91]. Furthermore, in vitro microtuber production in *Solanum tuberosum* was significantly greater in cryo-derived shoots than in the control [91].

Evaluating the field performance of cryo-derived plants of three *Chrysanthemum × grandiforum* chimeric cultivars Lady Orange, Lady Salmon, and Richmond, Kulus et al. [80] found that some cryo-derived plants had shorter internodes and shorter and/or narrower leaves than the control plants. The inflorescences of Lady Salmon opened slower, but faded faster than the control. However, flower traits, including color, diameter, fresh weight, and length of ray florets, were similar between the cryo-derived plants and the control in all three cultivars.

### 5.3. Reintroduction of Cryo-Derived Plants to Nature

*Castilleja levisecta,* a hemiparasitic herbaceous plant, naturally inhabits British Colombia, Canada and the USA. This species is currently listed as an endangered plant in Canada and the USA. Recently, a cryopreservation method was described for this plant [32]. Cryopreserved plants were successfully acclimated in greenhouse conditions. Acclimatized plants were reintroduced to their natural habitats in Canada and 21% of the reintroduced plants survived the transit from lab to the natural habitat and also showed flower development [32].

Hill’s thistle (*Cirsium hillii*) is also listed as a threatened species in Canada and its populations are restricted to alvars of Southern Ontario. Cryopreservation was applied for preserving germplasm of Hill’s thistle and cryopreserved plants were reintroduced to their natural habitats [25]. Field performances, including survival, vegetative growth, and plant developments, over 10 months were comparable or even better than the micropropagated plants (the control), although site-specific differences in the percent flowering and amount of indole amines compounds were observed among the plants [25].

These two studies provide a paradigm for the use of cryopreservation for the long-term preservation of plants at risk and their reintroduction in natural habitats.

## 6. Conclusions and Perspectives

Significant progress was made in the development of plant cryopreservation technology. A wide range of genetically diverse plants that are propagated sexually through seeds and vegetatively through shoot tips have now been successfully cryopreserved. The reintroduction of plants from cryobanks into natural habitats and assessments of field performance, including growth, reproduction, and adaptation to natural environments, was also achieved in many species. The plants from cryopreserved sources survived as well as those from non-cryopreserved tissues when established in vivo conditions. Overall, morphologies, vegetative growth, and reproduction of the cryopreserved plants were comparable to those of the non-cryopreserved ones. However, certain changes in DNA methylation were detected in the regenerants recovered after cryopreservation in some plant species. Some of these changes were attributed to in vitro culture processes, particularly in the cases of embryogenic tissues. The reversible epigenetic mechanism indicates that DNA methylation is temporary and plants can revert to normal DNA status when in vitro cryopreserved plants are established in the field conditions. Therefore, DNA methylation was not closely related to genetic variations in cryopreserved plants. Although the genetic variation was detected in the regenerants recovered after cryopreservation in some plant species, such variations were small in locus and low in frequency, and some of the variations were attributed to in vitro culture processes. Analyses by FCM and molecular markers proved that overall genetic stability was maintained in the regenerants recovered after cryopreservation in many of the plant species. Therefore, cryopreservation can maximally maintain genetic stability of cryo-derived plants compared to other traditional methods. Cryopreservation of transgenes provides a safe and reliable strategy for the long-term preservation of transgenic plant materials. Cryopreservation processes do not adversely affect the metabolic stability of plants of cryogenic origin and biochemical profiles are also maintained in the regenerated progeny. In addition, cryopreservation can preserve transgenes in the transformed materials.

Nevertheless, manipulations of in vitro stock cultures and cryogenic procedures are still needed to ensure genetic stability in the cryo-derived plants, and assessments of epigenetic and genetic stability in cryo-derived plants are necessary before any cryopreservation protocols are used for establishing cryobanks (Figure 1). Manipulations of in vitro stock cultures can be performed by shortening the time periods from establishing in vitro stock cultures to implementing cryopreservation as much as possible, and minimizing the use of plant growth regulators for the maintenance of in vitro stock cultures and post-culture of cryopreserved samples for plant recovery (Figure 1). Manipulations of cryoprocedures can be performed by avoiding or minimizing the use of toxic substances, such as DMSO, polyethylene glycol, and glycerol (Figure 1). Due to the low fraction of the genome screened in the analysis of genetic fidelity using available molecular markers, new methods are needed for broader screening of the genome in the regenerants recovered after cryopreservation. Next generation sequencing provides an ultra-high throughput platform by which the sequence of the entire genome of DNA or RNA can be determined and epigenetic variations can be detected, thus providing a solid evidence of epigenetic and genetic fidelity in the regenerants recovered after cryopreservation.

In conclusion, the process of cryopreservation seems to preserve the genetic make-up of plants and the plants regenerated from cryopreserved tissues demonstrate the ability to adapt through transient adaptation and through DNA methylation. It would be interesting to investigate the impact of stress ameliorating treatments before, during, and after cryotreatments at the biochemical and molecular level. Stress mitigation during the culture process prior and after vitrification may further improve the cryopreservation technology and reduce incidences of genetic and epigenetic variations.

## Figures and Tables

**Table 2:** Some examples from the past decade of genetic integrity assessments by molecular markers and FCM in regenerants recovered after cryopreservation.

Plant Species	Explants	Cryopreservation Method *	Molecular Markers **	Polymorphism (%)	Causes	Reference
*A* *bies*	Embryogenic cells	Vitri	RAPD	Not specified	Cryoprocedures and in vitro culture	[83]
*Actinidia chinensis* var. *deliciosa*	Shoot tips	Drop-vitri	AFLP and ISSR	None		[71]
*Allium cepa* var. *aggregatum*	Shoot tips	Drop-vitri	AFLP and ISSR	None		[99]
*Allium sativum*	Shoot tips	Vitri	SSR and FCM	None		[71]
*Arachis glabrata*	Leaflets	Drop-vitri	RAPD	0–3.4	Cryoprocedures	[119]
*Asparagus officinalis*	Rhizome buds	Encap-dehy	EST-SSR and FCM	None		[98]
*Bacopa monnieri*	Shoot tips	Vitri	RAPD	None		[106]
*Carica papaya*	Shoot tips	Vitri	RAF	0–0.7	Genotypes and cryoprocedures	[66]
*Chrysanthemum* × *grandiforum*	Shoot tips	Encap-dehy	ISSR	0–2	Genotypes and cryoprocedures	[80]
			RAPD	0–7.8		
			FCM	None		
*Chrysanthemum* × *morifolium*	Shoot tips	Encap-dehy	AFLP	40.1	Sucrose preculture	[85]
			RAPD	5.78		
		Drop-vitri	SSR	None		[82]
			FCM	None		
		Drop-vitri	ISSR and RAPD	None		[93]
*Cynara scolymus*	Shoot tips	Vitri	FCM	None		[108]
*Hedeoma todsenii*	Shoot tips	Encap-dehy and Encap-vitri	Microsatellite	5.36–13.04	Genotypes and cryoprocedures	[84]
			SRAP	4.55–20.45	Genotypes and cryoprocedures	
*Lotus tenuis*	Adventitious buds clusters	Vitri	ISSR	63	Cryoprocedures	[120]
*Malus* spp.	Shoot tips	Encap-dehy	ISSR	None		[101]
		Drop-vitri or Encap-dehy	ISSR and RAPD	None		[102]
*Mentha* × *piperita*	Shoot tips	Drop-vitri	RAPD	30–40	Genotypes and cryoprocedures	[121]
			RAPD	1–20	Genotypes and cryoprocedures	[86]
		Encap-dehy	RAPD	13–76	Genotypes and cryoprocedures	
			AFLP	0–85.7	Genotypes, cryoprocedures, and in vitro culture	[87]
			RAPD	0–62		
			AFLP	2.65	Sucrose preculture and encapsulation	[70]
			RAPD	None		
*Musa* spp.	Sucker meristems	Vitri	SSR	None		[122]
*Passiflora pohlii*	Nodal segments	Encap-vitri	ISSR and RAPD	None		[90]
		Vitri	ISSR and RAPD	None		
*Phaseolus vulgaris*	Seeds	Direct immersion into LN	SSR	None		[123]
*Picea abies*	Embryogenic tissues	Vitri	SSR	None		[124]
*Pinus nigra*	Embryogenic tissues	Slow-freezing	RAPD	None		[125]
*Pistacia vera*	Shoot tips	Vitri	RAPD	5.4	Cryoprotants and post-culture	[126]
*Pleione bulbocodioides*	Protocorm-like bodies	Vitri	ISSR	None		[96]
*Rabdosia rubescens*	Shoot tips	Encap-dehy	SRAP	0.01	Cryoprocedures	[105]
			FCM	None		
*Saccharum* spp.	Shoot tips	Drop-vitri	ISSR	1.5	Cryoprotection	[127]
*Solanum tuberosum*	Shoot tips	Vitri	AFLP and ISSR	None		[79]
		Drop-vitri	ISSR and RAPD	None		[91]
		Encap-vitri				
*Thymus lotocephalus*	Shoot tips	Drop-vitri	RAPD	0.06	Cryoprocedures	[128]
*Torenia fournieri*	Shoot tips	Drop-vitri	ISSRFCM	None		[95]
*Triticum aestivum*	Calli	Dehy	ISSR	None		[129]
			REMAP	0.3	Cryoprocedures	
*Vaccinium corymbosum*	Shoot tips	Drop-vitri	ISSR and RAPD	None		[103]
	Adventitious buds	Drop-vitri	ISSR and RAPD	None		[104]
*Vitis* spp.	Shoot tips	Drop-vitri	ISSR and RAPD	None		[17]
*Wasabia japonica*	Shoot tips	Vitri	AFLP	0.27–2.2	Cryoprocedures	[67]
*Ziziphus jujuba*	Shoot tips	Drop-vitri	FCM	None		[107]

* Dehy, dehydration; Drop, droplet; Encap, encapsulation; LN, liquid nitrogen; Vitri, vitrification. ** AFLP, amplified fragment length polymorphism; EST-SSR, expressed sequence tags-simple sequence repeats; FCM, flow cytometry; ISSR, inter-simple sequence repeats; SRAP, sequence-related amplified polymorphism; SSR, simple sequence repeats; RAF, randomly amplified DNA fingerprinting; RAPD, random amplified polymorphic DNA; REMAP, retrotransposon-microsatellite amplified polymorphism.

## Abbreviations

AFLP	Amplified fragment length polymorphism
DMSO	Dimethyl sulfoxide
FCM	Flow cytometry
HPLC	High performance liquid chromatography
ISSR	Inter-simple sequence repeats
LN	Liquid nitrogen
MSAP	Methylation sensitive amplified polymorphism
RAPD	Random amplified polymorphic DNA
ROS	Reactive oxygen species
SSR	Single sequence repeats
SRAP	Sequence-related amplified polymorphism
